# Partially substrateless microchannels for direct monitoring of interfacial dynamics in hydrophobic surfaces

**DOI:** 10.1038/s44172-025-00386-6

**Published:** 2025-03-13

**Authors:** Ellen Bold, Sebastian Zimmermann, Clarissa Schönecker, Egbert Oesterschulze

**Affiliations:** 1 Rhineland-Palatinate Technical University (RPTU) Kaiserslautern, Department of Physics, Physics and Technology of Nanostructures, Erwin-Schrödinger Str. 46, Kaiserslautern, 67663 Germany; 2 Rhineland-Palatinate Technical University (RPTU) Kaiserslautern, Department of Mechanical Engineering, Microfluidics, Gottlieb-Daimler Str. 46, Kaiserslautern, 67663 Germany

**Keywords:** Mechanical engineering, Fluid dynamics

## Abstract

Superhydrophobic and liquid-infused surfaces are the most prominent techniques to achieve drag reduction in microchannels. However, they have specific drawbacks such as costly fabrication of complex and mechanically sensitive surfaces, surfaces susceptible to lubricant abrasion or involve hazardous chemicals. We present a partially substrateless microchannel whose upper wall features a large no-shear air/water meniscus at atmospheric pressure. On this wall, a self-assembled monolayer of hydrophobic alkyl silane was bonded covalently. Flow experiments reveal a drag reduction of up to 25% although only 4% of the wall fulfils the no-shear condition. These experiments demonstrated long-term stability and self-healing properties. Furthermore, White Light Interferometry (WLI) was used for direct monitoring of interfacial dynamics. By optical investigation of the full meniscus topography the contact-free evaluation of the spatially resolved static pressure distribution was possible. Conducted numerical simulations are in good agreement with the experimental findings and illustrate the drag reduction mechanism.

## Abstract (web summary)

Ellen Bold and colleagues address the high flow resistance in microchannels with shear-free walls. A partially substrateless microchannel with a shear-free meniscus wall was implemented, showing up to 25% drag reduction, long-term stability and self-healing properties.

## Introduction

One of the most prominent biomimetic concepts is the technical realisation of the ability of natural surfaces to repel water. Inspired by the natural hierarchical surface structures of the lotus leaf^1,2^, this fascinating manifestation of so-called superhydrophobic surfaces (SHSs) has attracted considerable interest in recent years. Artificially manufactured SHSs currently offer a multitude of potential applications including anti-icing^3–5^ and anti-biofouling^6,7^. Another important field of application is the fluid flow through conventional microchannel systems that are prone to strong drag owing to the no-slip condition on the solid microchannel walls. Here, the integration of SHS provides a method to achieve a significant reduction of drag rendering slip on the surfaces possible^8^.

The creation of artificial SHSs was achieved through the combination of two main principles^9^: On the one side the chemical modification of substrates through deposition of per- or polyfluoroalkyl substances (PFAS) results in the formation of surfaces with exceptional water-repellent properties^10,11^. The degradation of these substances presents a significant risk due to their persistence in the environment and their tendency to bioaccumulate in living organisms posing dangerous health risks^12–15^. On the other side the enclosure of air in small cavities of a rough surface featuring hierarchical micro- and nanostructures demonstrated reduction of drag. A variety of structural configurations^16–20^ were presented and demonstrate that while maintaining the surface of the wall in the Cassie-Baxter (CB) state, the increased air-to-liquid surface ratio allows the liquid to slip with reduced resistance^21^. For comparing and evaluating the drag reduction effects of different sample structures, the concept of the slip length *λ* was introduced. It is defined as the distance beyond the interface at which the velocity profile linearly extrapolates to zero^22^. The presence of these small air pockets results in a notable slip length, reaching up to several micrometres compared to nanometres for hydrophobic coated surfaces^23,24^.

However, such interfaces are susceptible to high external pressures, material irregularities, vibrations or forced condensation in these cavities, causing the menisci to depin or collapse^25,26^. These degradation processes are accompanied by the irreversible transition from the CB state into the sticky Wenzel state causing the loss of drag reduction capability of such surfaces^27^. Although the CB state stability can be improved by employing smaller surface structures, e.g. nanostructures, this typically leads to only small slip lengths, as the latter scales with the dimension of the surface structures^9^. Moreover, the robustness of these complex nano- and microstructures against abrasion is a crucial factor, as mechanical failure through e.g. bending of the microstructure also results in the depletion of enclosed air^9,28,29^.

Entrapping low viscosity lubricants instead of air can significantly reduce or even avoid some of the aforementioned stability issues. The continuously increasing research focus on liquid-infused surfaces (LIS) is driven by their resilience against high external pressure and also by their self-healing capability^30,31^. Meanwhile, particular LIS surfaces feature a drag reduction capability comparable to SHS^32^. However, elevated viscous interface interaction may impede the drag-reducing performance of these surfaces^22^. Additionally, it leads to shear-induced drainage, whereby the low viscosity lubricant is partially dragged out of the surface textures, resulting not only in the contamination of the flowing fluid and its environment but also in the partial or complete loss of drag reduction^33,34^.

The approaches effecting drag reduction in microchannels discussed so far have used completely closed channel systems. Karatay et al. introduced a microchannel with a periodic set of openings in one of the microchannel walls, each of which is spanned by a microbubble^35^. This so-called bubble mattress allows the application of an external air pressure changing the topography of the menisci, including the angle at which they are pinned to the edges of the surface structures. A maximum drag reduction of 23% was achieved for the optimum pinning angle of 10^∘^ with a surface area fraction of the gas/fluid interface of about 8% but only by active control of the micro-bubbles applying an external pressure.

This paper presents a microchannel in which a fully closed, no-slip microchannel has been modified to become partially substrateless. This was achieved by introducing a single freely accessible large air/fluid meniscus, best described as a no-shear boundary surface at atmospheric pressure. This set-up is advantageous because no fragile micro- or nanostructures are required and instead of PFAS, covalently bonded self-assembly monolayers of alkyl silane are used as hydrophobic coating. It shows a very good stability of the CB state and a comparatively large drag reduction effect. Drag reduction of this system is investigated in flow experiments. We propose to evaluate the spatially resolved static pressure distribution by optical imaging of the complete meniscus with a White Light Interferometer (WLI). The measured meniscus topography was used as input for numerical simulations to investigate the impact of both the variation of the cross-sectional area and the no-shear boundary condition at the meniscus surface on drag reduction.

## Results

In contrast to existing drag reducing microchannel concepts that use complex micro- and nanostructured walls as surfaces with increased slip length, the basic idea of the partially substrateless microchannel (PSC) is to replace part of the solid channel walls by an air/liquid meniscus. This is achieved simply by removing part of the channel walls and spanning menisci in the open frames during flow operation. Various geometries of the frame structure are possible, such as a periodic array of apertures or slits. However, to investigate the basic principle of a PSC, we have focused here on a single rectangular shaped slit with a large dimension in the flow direction and a small dimension in the perpendicular direction.

### Experimental setup of the microchannel and analyzing methods

**Fig. 1:**
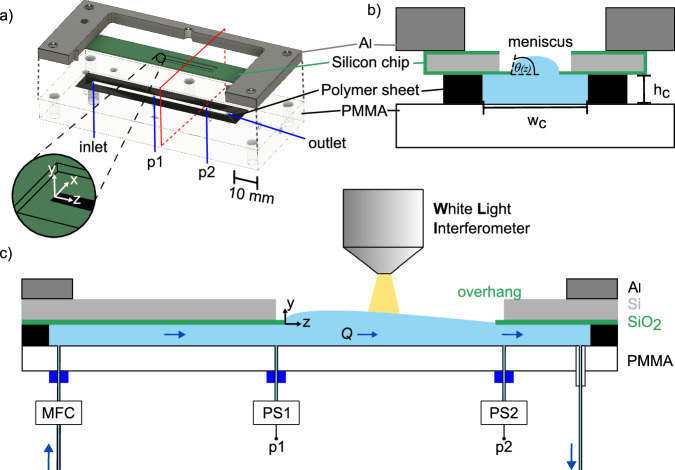
Setup for optical and pressure measurements. **a** Explosive view of the scheme of the stacked microchannel device: For the sake of clarity the front bar of the aluminium plate and the screws pressing the stack together are not shown. **b** Schematic cross section of the device at the position marked by the red rectangle in (**a**) during operation. It shows the cross-sectional geometry of the microchannel device with *θ*(*z*) as the local pinning angle of the meniscus at the silicon dioxide overhang structure. **c** Scheme of the cross-section of the microchannel device, taken along the direction of flow. The flow rate *Q* is preset by a flow controller (MFC), while the pressure drop is obtained as the difference between the measured pressures at PS1 and PS2. The complete 3D meniscus shape is imaged with the WLI from the top by stitching. Panels (**b**) and (**c**) not to scale.).

The key component of our microchannel is a macroscopically structured silicon dioxide (SiO_2_) coated silicon chip with a 25 mm long and 0.23 mm wide slit. During plasma etching of silicon to form the macroscopic slit, the SiO_2_ layer is slightly underetched forming a SiO_2_ overhang structure of 3 μm width (see Fig. 1). Processing is finished by coating the SiO_2_ layer with a hydrophobic OTCS (Octadecyltrichlorosilane) self assembled monolayer (Details in “Methods” and Section S1 of Supplementary Information).

Our modular microchannel device consists of a stack of a flat PMMA (Polymethylmethacrylate) base plate (*l* = 87.5 mm, *w* = 22.7 mm, *t* = 6 mm), a polymer sheet (type: ORACAL®751C High Performance Cast, *w*_s_ = 12.5 mm, *l*_s_ = 85 mm, *h*_c_ = 0.176 mm) with a rectangular opening (*w*_c_ = 2.5 mm, *l*_c_ = 65 mm) defining the channel geometry (*w*_c_, *l*_c_) as well as its height (*h*_c_), and the rectangular silicon chip as the top plate. To ensure reliable sealing of the channel, we placed a 5 mm thick aluminium plate on top of the silicon chip and screwed it to the PMMA base plate. The rectangular opening in the aluminium plate was chosen to be larger than that in the silicon chip in order to have optical access to the full air/water meniscus during flow operation with the White Light Interferometer (WLI, Zygo NexView NX2). Figure 1 shows schemes of a) the explosive view of the device and b) a profile of the device at the cross sectional area marked by the red rectangle in a) showing the meniscus formed in the slit of the silicon top plate during operation with its local pinning angle *θ*(*z*). For later reference a coordinate system was implemented with its origin centred at the beginning of the slit opening with the *z*-axis in flow direction (Fig. 1a).

Additionally, a conventional microchannel device with a rectangular cross-section of the same dimensions (*w*_c_, *h*_c_) (Fig. 1b) and no-slip walls was fabricated. It is used as reference microchannel device (RC) during flow experiments discussed in Section “Pressure sensor measurements”.

A pressure-operated system was used to establish a stable and continuous flow over an extended period of time (Fig. 1c). The flow rate was regulated and measured by a flow controller (MFC) (Premium Coriolis Flow Sensor BFS2, Elveflow) connected to a liquid reservoir. To establish and stabilise a laminar flow in the slit region an inlet length of 3 mm and an outlet length of 1 mm were considered^36^. A distance of 1 mm was kept between the two contact flanges of the pressure sensors (PS1 and PS2 in Fig. 1c) (Microfluidic Pressure Sensor MPS0, Elveflow) with respect to the beginning and end of the slit to prevent any disturbance of the meniscus. Prior to measurements, the system was filled with deionised water (density *ρ* = 998.207 kg m–3, dynamic viscosity *η* = 0.001 Pa ⋅ s, surface energy *σ* = 0.072 J m–2), including the channel and pressure sensor tubing. This avoids the occurrence of air bubbles becoming trapped at the pressure sensor to channel junction. To determine local profiles of the meniscus, its entire surface topography was imaged by stitching images of the WLI at different flow rates while simultaneously measuring the pressure drop with the pressure sensors.

### Pressure sensor measurements

Flow experiments of the RC were conducted varying *Q* and simultaneously measuring the pressure drop Δ*p* = *p*_2_ − *p*_1_ with the pressure sensors PS2 and PS1 (Fig. 1c) along the flow direction. For the flow rates between $250\,\mu {{\rm{l}}}\,\min$-1 and $1500\,\mu {{\rm{l}}}\,\min$-1 employed in the experiments the Reynolds numbers are between 2 and 10, indicating laminar flow conditions irrespective of the RC or PSC microchannel. Therefore, we can apply the Poiseuille theory for laminar flow of an incompressible flow in the RC to evaluate the pressure drop under no-slip conditions^37^:

1 $$\Delta {p}_{{{\rm{RC}}}}^{{{\rm{theo}}}}=K\cdot \frac{12\eta {l}_{{{\rm{c}}}}}{{h}_{{{\rm{c}}}}^{3}{w}_{{{\rm{c}}}}}\cdot Q.$$

Assuming that all four walls are of the no-slip boundary type, the parameter *K* is:

2 $$K=1-\mathop{\sum }\limits_{n=1}^{\infty }\frac{1}{{(2n-1)}^{5}}\cdot \frac{192}{{\pi }^{5}}\cdot \frac{{h}_{{{\rm{c}}}}}{{w}_{{{\rm{c}}}}}\tanh \left((2n-1)\frac{\pi }{2}\frac{{w}_{{{\rm{c}}}}}{{h}_{{{\rm{c}}}}}\right).$$

**Fig. 2:**
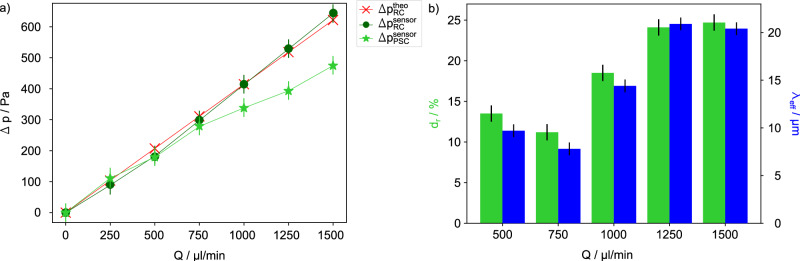
Influence of PSC on the pressure drop. **a** Pressure drop Δ*p* versus flow rate *Q* for the RC calculated using Eq. (1) ($\Delta {p}_{{{\rm{RC}}}}^{{{\rm{theo}}}}$ red cross) and measured with pressure sensors ($\Delta {p}_{{{\rm{RC}}}}^{{{\rm{sensor}}}}$ dark green circle and $\Delta {p}_{{{\rm{PSC}}}}^{{{\rm{sensor}}}}$ green star for the PSC). The pressure drop of the RC exceeds that of the PSC by Δ*p* = 477.7  ±  30 Pa for the maximum flow rate of $Q=1500\,\mu {{\rm{l}}}\,\min$-1. **b** The progression of the drag reduction factor *d*_r_ (light green) and the slip length *λ*_eff_ (blue) with increasing *Q* for the PSC. Vertical error bars of  ±  30 Pa are given by the resolution of the pressure sensors (see Section S3 Supplementary Information).

The measured pressure drop $\Delta {p}_{{{\rm{RC}}}}^{{{\rm{sensor}}}}$ of the RC (Fig. 2 dark green circle) and the theoretically determined pressure drop $\Delta {p}_{{{\rm{RC}}}}^{{{\rm{theo}}}}$ (Fig. 2 red cross) calculated with Eq. (1) are in excellent agreement showing the expected linear dependence of $\Delta {p}_{{{\rm{RC}}}}^{{{\rm{sensor}}}}$ with *Q*.

Performing now the same flow experiments with the PSC shows also a continuous increase of the pressure drop $\Delta {p}_{{{\rm{PSC}}}}^{{{\rm{sensor}}}}$ with *Q* (Fig. 2 light green star). However, $\Delta {p}_{{{\rm{PSC}}}}^{{{\rm{sensor}}}}$ is always smaller compared to $\Delta {p}_{{{\rm{RC}}}}^{{{\rm{theo}}}}$ and even more important is that the difference $\Delta {p}_{{{\rm{RC}}}}^{{{\rm{theo}}}}-\Delta {p}_{{{\rm{PSC}}}}^{{{\rm{sensor}}}}$ increases with increasing *Q* indicating that the drag reduction performance of the PSC improves significantly with increasing *Q*. To quantify this behaviour we applied the definition of the drag reduction factor *d*_r_ introduced by Ou et al.^38^:

3 $${d}_{{{\rm{r}}}}=\frac{\Delta {p}_{{{\rm{RC}}}}^{{{\rm{theo}}}}-\Delta {p}_{{{\rm{PSC}}}}^{{{\rm{sensor}}}}}{\Delta {p}_{{{\rm{RC}}}}^{{{\rm{theo}}}}},$$

comparing $\Delta {p}_{{{\rm{PSC}}}}^{{{\rm{sensor}}}}$ with $\Delta {p}_{{{\rm{RC}}}}^{{{\rm{theo}}}}$ the pressure drop obtained with Eq. (1). This allows to calculate the effective slip length *λ*_eff_, representing the mean slip of the microchannel, as follows^34^:

4 $${\lambda }_{{{\rm{eff}}}}=\frac{{h}_{{{\rm{c}}}}{d}_{{{\rm{r}}}}}{3-4{d}_{{{\rm{r}}}}}.$$

As shown in the bar graph (Fig. 2b) both *d*_r_ and *λ*_eff_ increase with *Q* to excellent values of 24.7 % and 20.4 μm, respectively for a maximum flow rate of ${Q}_{\max }=1500\,\mu {{\rm{l}}}\,\min$-1. Although a fairly large slit shaped meniscus of 0.085 *γ* × 9.26 *γ* with dimensions measured in units of the capillary length $\gamma =\sqrt{\sigma /(\rho g)}$ (*γ* = 2.73 mm for water) was used, it replaces only 4% of the solid/liquid interface in the PSC by the air/liquid meniscus interface. For the explanation of the increasing *d*_r_ and *λ*_eff_ with *Q*, at least two effects are decisive: for increasing *Q* the meniscus is blown up forming both a larger cross sectional area of the PSC and simultaneously also a larger surface area of the meniscus with no-shear boundary condition.

When *Q* exceeds a flow rate of about ${Q}_{\max }=1500\,\mu {{\rm{l}}}\,\min$-1, the local pressure at the beginning of the slit will surpass the Laplace pressure drop across the meniscus, resulting in its rupture. The critical pressure drop that the meniscus can withstand is given by $\Delta {p}_{{{\rm{YL}}}}^{{{\rm{crit}}}}\,=\,2\sigma \sin ({\theta }_{{{\rm{crit}}}})/{w}_{{{\rm{c}}}}$ where *θ*_crit_ is the critical contact angle (see Section S9 in Supplementary Information). For our channel geometry we find $\Delta {p}_{{{\rm{YL}}}}^{{{\rm{crit}}}}\,=\,573\,{{\rm{Pa}}}$ which agrees to the pressure value of about 600 Pa measured with the pressure sensor PS1 for $Q=1500\,\mu {{\rm{l}}}\,\min$-1.

**Fig. 3:**
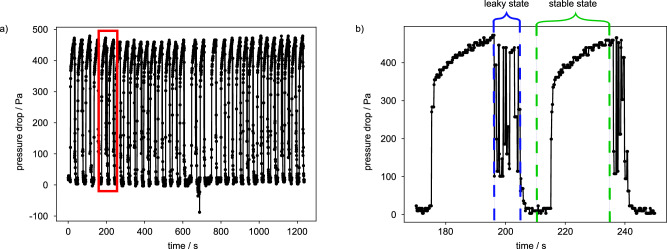
Long term stability of PSC. **a** The microchannel was subjected to repeated measurements, with *Q* switched between $0\,\mu {{\rm{l}}}\,\min$-1 and $> 1500\,\mu {{\rm{l}}}\,\min$-1, in order to provoke rupture of the meniscus interface. **b** For a closer look at the details in (**a**) we show in (**b**) a zoom image taken from the red box in (**a**). The plot in (**b**) illustrates the transition from a leaky state, where the pressure fluctuates due to the rupture of the meniscus, to a stable meniscus state accompanied by flow through the microchannel. The meniscus was recovered after *Q* had been reduced to $0\,\mu {{\rm{l}}}\,\min$-1 for a few seconds.

The failure of drag reduction properties in the case of SHS is caused by an irreversible CB-to-Wenzel transition, whereas the underlying mechanism of the PSC failure is meniscus rupture. However, the PSC exhibits self-healing properties. This was investigated by varying *Q* as a periodic square wave signal. Employing an amplitude *Q* that exceeds ${Q}_{\max }$, causes the rupture of the meniscus and thus the interruption of the microchannel flow (see Fig. 3). However, when *Q* is set to $0\,\mu {{\rm{l}}}\,\min$-1 for only a few seconds, self-healing of the meniscus was observed resulting in regular flow through the PSC with the same favourable *d*_r_ value.

In the context of the flow experiments, we would also like to address the temporal meniscus stability. Our PSC was operated for 24 h presetting the flow rate to 1000 $\,\mu {{\rm{l}}}\,\min$-1 to investigate in particular the temporal stability of the meniscus and the resultant flow properties. During this period, the continuously measured pressure drop was stable and small fluctuations due to temperature variations lead to a standard deviation of 3.5% (see Fig. S6 in Supplementary Information). These results underline that the meniscus is stable under the imposed flow rate and keeps its stability over time.

### Optical investigation of the pressure distribution with WLI

The two-point pressure measurement used so far is insufficient to investigate the interfacial dynamics in the PSC. Therefore, we propose to first resolve the meniscus topography optically and deduce the pressure distribution in the PSC from the optical data. For this purpose, a WLI was used due to its non-contact and non-intrusive properties and in particular its excellent *z* − resolution below 1 nm. Imaging was achieved by stitching approximately 100 image frames with an overlap of 80%. The meniscus topography is later used as input geometry for numerical simulation to study the impact of the shear free boundary condition on the flow behaviour (see Section “Numerical study”).

**Fig. 4:**
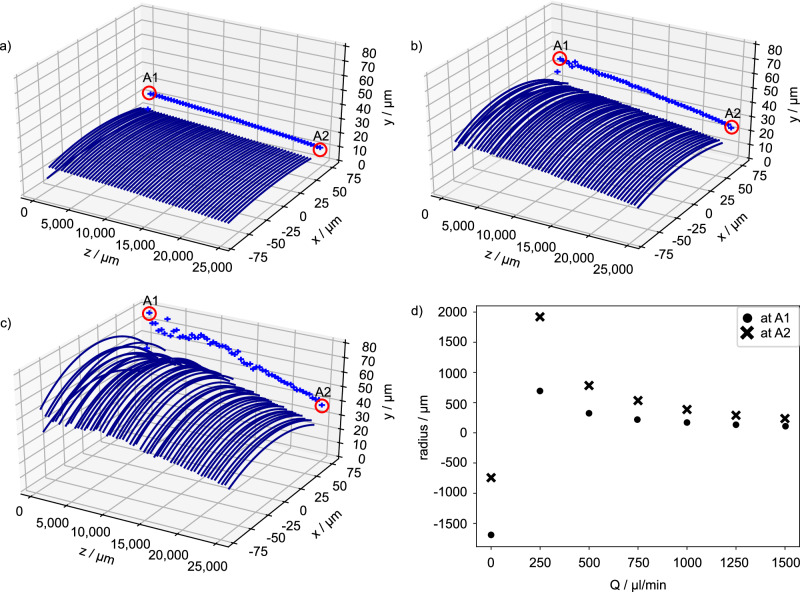
Evolution of meniscus profiles with increase in flow rate. Meniscus profiles extracted from WLI measurements of the meniscus surface perpendicular to the flow direction at an separation distance of Δ*z* = 0.5 mm within the imaging constraints (more details in Section S8 in Supplementary Information). The maximum profile height *h*(*z*) was additionally projected onto the *y* − *z* plane (blue crosses). At flow rates *Q* of (**a**) $500\,\mu {{\rm{l}}}\,\min$-1 and (**b**) $1000\,\mu {{\rm{l}}}\,\min$-1 the height of meniscus profiles reveal a linear decrease in flow direction. **c** At the highest flow rate of $Q=1500\,\mu {{\rm{l}}}\,\min$-1 the height of the meniscus profiles shows an almost linear decrease with small deviations. **d** The figure illustrates the development of the radius *R*_⊥_ at positions A1 and A2 for all flow rates *Q*.

For the sake of clarity we have shown in (Fig. 4a–c) for the flow rates of 500, 1000 and $1500\,\mu {{\rm{l}}}\,\min$-1 only 50 profiles of the meniscus perpendicular to the flow direction each with a separation distance of Δ*z* = 0.5 mm. The meniscus profile obtained for each *Q* can be found in Section S4 in the Supplementary Information. From these profiles we can determine the local radius *R*(*z*) of curvature, the height *h*(*z*) of each profile above the silicon dioxide layer and the pinning angle *θ*(*z*) of the meniscus. The height *h*(*z*) of each profile was projected onto the *y* − *z* plane in (Fig. 4a–c) and shows a linear drop along the slit. Only for a flow rate of $1500\,\mu {{\rm{l}}}\,\min$-1 (see Fig. 4c) the profiles show small fluctuations primarily at the beginning. Additionally, the local pinning angle *θ*(*z*) extrapolated from the local profiles to the surface shows also a linear behaviour (see Section S5 in the Supplementary Information).

From meniscus profiles taken at the beginning (A1 at *z* ≈ 1 mm) and the end (A2 at *z* ≈ 24.8 mm) (see Fig. 4a–c) additionally marked with red circles), the radius of curvature perpendicular to the flow direction *R*_⊥_ was evaluated for each flow rate (Fig. 4d). When the flow rate was switched off, the stationary meniscus surface was concave, which is due to the hydrophobic OTCS coating of the overhang structure. The discrepancy in *R*_⊥_ at the beginning (−1702 μm) and end (−747 μm) of the slit may be attributed to the fact that our method to determine the radius of curvature perpendicular to the flow direction for a meniscus pinned at the upper edge of the overhang structure is not appropriate here. In fact, we observed with the WLI, that the location of the pinning meniscus repeatedly jumps between the lower and upper edge of the slit. If the flow rate exceeds a certain limit of approx. $100\,\mu {{\rm{l}}}\,\min$-1, a convex shape with a positive radius of curvature is obtained. For further increasing flow rates, the radius of curvature at the end of the meniscus always exceeds that at its beginning owing to the negative pressure gradient in flow direction.

With the optically evaluated local radius of curvature of the meniscus, the static pressure drop *p*_stat_(*z*) across its interface at any given position *z* can be determined using the Young-Laplace equation^39^:

5 $${p}_{{{\rm{stat}}}}(z)=\Delta {p}_{{{\rm{YL}}}}(z)=\sigma \left(\frac{1}{{R}_{\perp }}+\frac{1}{{R}_{\parallel }}\right),$$

where *σ* is the surface tension of water and *R*_⊥_ = *R*_⊥_(*z*) and *R*_∥_ = *R*_∥_(*z*) are the local principal radii of curvature at position *z*. We can simplify Eq. (5) neglecting 1/*R*_∥_, owing to the linear height variation in *z* direction, and yield:

6 $${p}_{{{\rm{stat}}}}(z)\approx \frac{\sigma }{{R}_{\perp }}.$$

**Fig. 5:**
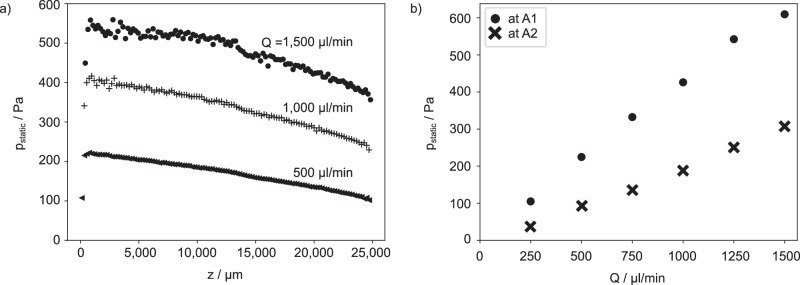
Optically determined local static pressure along the meniscus. **a** *p*_stat_(*z*) extracted from WLI data along the flow direction *z* in steps of Δ*z* = 0.5 mm for *Q* values of: $500\,\mu {{\rm{l}}}\,\min$-1, $1000\,\mu {{\rm{l}}}\,\min$-1, and $1500\,\mu {{\rm{l}}}\,\min$-1. **b** The static pressure was determined for all flow rates at the two positions marked with red circles in Fig. 4 close to the beginning (A1) and end (A2) of the meniscus.

The static pressure decreases along *z* whereby its negative gradient increases with *Q* (see Fig. 5).

As will be presented in Section “Comparison of the different evaluation methods”, the pressure drop calculated from the optical WLI measurements (orange triangle), between A1 and A2, was found to be 458.4 ± 2 Pa for $Q=1500\,\mu {{\rm{l}}}\,\min$-1, resulting in a drag reduction of approximately 25%. This value is consistent with the sensor measurements, considering the pressure drop over the distance PS1 to A1 and A2 to PS2 applying Eq. (1).

In contrast to the two-point sensor measurements, the entire topography of the meniscus can be resolved using the WLI. The topographic data provides information about the static pressure profile *p*_stat_(*z*) and the local pinning angle *θ*(*z*) of the whole meniscus. This allows us to investigate the origin of the drag reduction as will be discussed in Section “Numerical analysis of the mechanism of drag reduction”.

### Numerical study

While a considerable number of studies have investigated the potential drag reduction of fluid flow along surfaces textured with longitudinal slots, the majority have either assumed that the interfaces are flat or investigated the case of a constant downstream meniscus deflection^40,41^. In contrast, Game et al.^42^ have developed a model that supports a slowly varying meniscus protrusion in the downstream direction, thereby allowing the consideration of highly protruded menisci at the point of entry and flat menisci at the end of the slit. However, in an effort to analyse the overall flow behaviour at the slit inlet and outlet with greater accuracy, it is necessary to also consider the conditions within the closed channel both prior to the start of the slit and after its end. This is why the authors chose to employ a fully numerical calculation model that incorporates optical measurements (see Section “Optical investigation of the pressure distribution with WLI”) of meniscus protrusion to capture the shape of the interface, allowing a local resolution of the velocity field within the channel.

The optically measured radii of the meniscus at A1 and A2 and its linear decrease between them was modelled by a cylinder inclined with respect to the open frame in the SiO_2_ layer. In order to account for the pinning of the meniscus at the rim of the SiO_2_ overhang structure, the shape of the meniscus was quadratically interpolated to zero between the start of the slit and A1, and between A2 and the end of the slit. The meniscus was assumed as a no-shear interface. A more detailed description of the modelling setup is presented in Methods (Section “Numerical setup”).

**Fig. 6:**
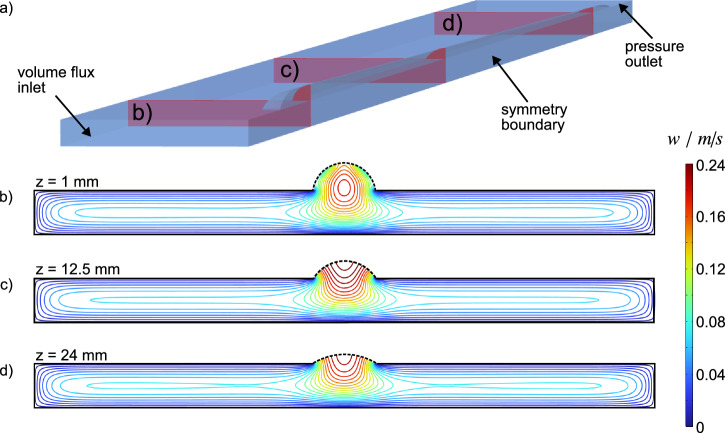
Fluid domain and velocity field contour-line plots. **a** Illustrates the half-width computational domain of the water-phase. Red planes indicate the (*x*, *y*)-cross-sections associated with the subsequent plots. Contour-lines for $Q=1500\,\mu {{\rm{l}}}\,\min$-1 with black dashed lines indicating the position of the shear-free curved air/fluid interface at the positions (**b**) *z* = 1.0 mm, (**c**) *z* = 12.5 mm and (**d**) *z* = 24.0 mm.

Figure 6a shows the computational domain of our numerical study for a volume flux of $1500\,\mu {{\rm{l}}}\,\min$-1. To illustrate the influence of the interface protrusion on the overall flow behaviour, (Fig. 6b–d) show contour line plots of the velocity field *w*(*x*, *y*) corresponding to the (*x*, *y*)-cross-sections marked as red planes in (Fig. 6a) distanced by 11.5 mm from each other. At *z* = 1 mm (Fig. 6b), the interface is almost maximally protruded, i.e. the channel cross-section is significantly expanded locally at the position of the slit. The position of the maximum velocity ${w}_{\max }$ along *z* depends on the magnitude of the meniscus curvature. Further downstream ${w}_{\max }$ shifts closer to the meniscus, where the influence of all no-slip walls is minimal (Fig. 6c, d).

A comparison of these maximum velocities shows, that at *z* = 1 mm we observe the relatively lowest ${w}_{\max }=0.18\,{{\rm{ms}}}-1$ of the three cases considered in Fig. 6. The reason is the enlargement of the local cross-section, since for a given volume flux, the mean velocity must decrease according to the conservation of mass. At *z* = 12.5 mm, i.e. halfway along the slit, we reach the highest maximum velocity (${w}_{\max }=0.24\,{{\rm{ms}}}-1$). This behaviour is similar to the principles governing flow over grooved surfaces^43^. However, at *z* = 24 mm, this value drops to ${w}_{\max }=0.19\,{{\rm{ms}}}-1$. Even though the channel narrows downstream, because of the decreasing interface protrusion, the flow already experiences the influence of the solid wall at the end of the groove.

**Fig. 7:**
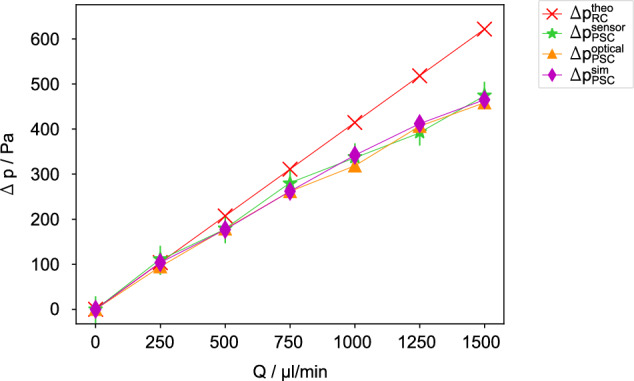
PSC shows a considerable reduction of pressure drop. Pressure drop (Δ*p* versus flow rate *Q* for RC calculated using Eq. (1) ($\Delta {p}_{{{\rm{RC}}}}^{{{\rm{theo}}}}$ red cross) and PSC data measured with pressure sensors ($\Delta {p}_{{{\rm{PSC}}}}^{{{\rm{sensor}}}}$ green star) and optically ($\Delta {p}_{{{\rm{PSC}}}}^{{{\rm{optical}}}}$ orange triangle) and simulated considering the meniscus protrusion ($\Delta {p}_{{{\rm{PSC}}}}^{{{\rm{sim}}}}$ magenta diamond) derived from the experimental data. It shows that the difference $\Delta {p}_{{{\rm{RC}}}}^{{{\rm{theo}}}}-\Delta {p}_{{{\rm{PSC}}}}^{{{\rm{i}}}}$, with *i* standing for sensor, optical and simulation data, increases with increasing *Q*.

For better comparability, we evaluated the static pressure at *z* = −1 mm and *z* = 26 mm, which are the positions of the pressure sensors in the experiment. The pressure of the RC obtained from simulations correspond to the analytically calculated pressure using Eq. (1). Figure 7 illustrates the numerically computed pressure drop for PSC ($\Delta {p}_{{{\rm{PSC}}}}^{{{\rm{sim}}}}$ magenta diamond), demonstrating that no significant differences are observable for low volume rates. However, an increasing drag reduction is evident for *Q* exceeding 1000 $\mu {{\rm{l}}}\,\min$-1.

### Comparison of the different evaluation methods

In Fig. 7 all pressure drop results of the PSC measured with pressure sensors ($\Delta {p}_{{{\rm{PSC}}}}^{{{\rm{sensor}}}}$ green star), evaluated from optically data ($\Delta {p}_{{{\rm{PSC}}}}^{{{\rm{optical}}}}$ orange triangle), and simulated considering the meniscus protrusion ($\Delta {p}_{{{\rm{PSC}}}}^{{{\rm{sim}}}}$ magenta diamond) are presented. The data demonstrate a strong correlation and a markedly lower pressure drop compared to the theoretically calculated value $\Delta {p}_{{{\rm{RC}}}}^{{{\rm{theo}}}}$ (red cross) of the RC.

Drag reduction *d*_r_ (Eq. 3) and slip length *λ*_eff_ (Eq. 4) are used to fully compare the data with each other. The data presented in Table 1 reveals a non-linear increase in both *d*_r_ and *λ*_eff_ with *Q*. The values of both *d*_r_ and *λ*_eff_ increase from 10% and 10 μm to 25% and 20 μm, respectively, demonstrating a significant drag reduction of the PSC compared to the RC. This finding is consistent with the results observed in the graphs of pressure drop versus flow rate in Fig. 7, where the slope representing the flow resistance, decreases for the PSC non-linearly with increasing flow rate. It is important to note that the validity of the simulated data requires the use of the complete optically measured meniscus shape as geometric input. This data can’t be retrieved if only the two-point pressure sensor data is available. Furthermore, the optical measurement technique is advantageous compared to sensor measurements, in particular if pressure drops smaller than the sensitivity threshold of the sensors of  ±30 Pa needs to be detected. This requires the measurement of a large radius of curvature, which is the domain of the WLI with its superior *z*-resolution. With only a single macroscopic slit we received a rather large slip length, in comparison to fully microstructured superhydrophobic surfaces^44,45^.

**Table 1:** Comparison of the drag reduction factor *d*_r_ (Eq. (3)) and the effective slip length *λ*_eff_ (Eq. (4)) as function of *Q* for sensor and optical measurements and the simulation data of the PSC

	sensor measurement		optical measurement		simulation	
*Q* / $\mu {{\rm{l}}}\,\min$-1	*d*_r_/%	*λ*_eff_ / *μ*m	*d*_r_/%	*λ*_eff_ / *μ*m	*d*_r_/%	*λ*_eff_ / *μ*m
500	13.5	9.7	13.9	10.1	14.6	10.7
750	11.2	7.8	15.9	11.9	15.7	11.8
1000	18.5	14.4	23.2	19.8	17.5	13.5
1250	24.1	20.9	21.6	17.9	20.5	16.6
1500	24.7	20.4	26.3	23.9	25.2	22.4

### Numerical analysis of the mechanism of drag reduction

Further simulations were conducted to examine the mechanism of drag reduction. *Q* was varied from 500 to 1500$\,\mu {{\rm{l}}}\,\min$-1 in steps of 250$\,\mu {{\rm{l}}}\,\min$-1 taking both the boundary condition at the air-water interface and the interface deflection in the slit area into account. The main findings are presented in Table 2.

**Table 2:** Influence of flow boundary condition and increase in surface area on pressure drop

$Q\,/\,\mu {{\rm{l}}}\,\min$-1	$\Delta {p}_{{{\rm{RC}}}}^{{{\rm{sim}}}}\,/\,{{\rm{Pa}}}$	$\Delta {p}_{{{\rm{RC,prot.}}}}^{{{\rm{sim}}}}\,/\,{{\rm{Pa}}}$	$\Delta {p}_{{{\rm{PSC}}}}^{{{\rm{sim}}}}\,/\,{{\rm{Pa}}}$
500	203	201 (~1.0%)	177 (~12.0%)
750	305	300 (~1.6%)	262 (~12.6%)
1, 000	408	397 (~2.7%)	342 (~13.9%)
1, 250	509	487 (~4.3%)	412 (~15.4%)
1, 500	612	568 (~7.2%)	465 (~18.1%)

Comparison of pressure-drops as a function of the flow rate *Q*, where $\Delta {p}_{{{\rm{RC}}}}^{{{\rm{sim}}}}$ is used as reference. Additionally, $\Delta {p}_{{{\rm{RC,prot.}}}}^{{{\rm{sim}}}}$ describes the pressure-drop along the channel, taking into account the increased channel cross-section as a consequence of the interface protrusion. Crucially, these interfaces are modelled as no-slip boundaries rather than no-shear, to emphasise the isolated impact of duct expansion. Lastly, $\Delta {p}_{{{\rm{PSC,prot.}}}}^{{{\rm{sim}}}}$ is the pressure-drop considering curved shear-free menisci, as investigated in our experiments. The values in brackets indicate the relative change in pressure drop compared to the previous column.

First, we simulated the pressure-drop $\Delta {p}_{{{\rm{RC}}}}^{{{\rm{sim}}}}$ for the reference sample over a downstream length of 27 mm with a flat top wall and no-slip boundary condition. Subsequently, based on the WLI measurements a three-dimensional geometric model of each interface (analogue to Section “Comparison of the different evaluation methods”) was used in the numerical model. To isolate the impact of the increased channel volume on the associated drag reduction, the protruded interfaces were first modelled as pure no-slip walls. The results are labelled as $\Delta {p}_{{{\rm{RC}}}}^{{{\rm{sim}}}}$ in Table 2. For comparatively low volume rates, such as $Q\le 1000\,\mu {{\rm{l}}}\,\min$-1, only a slight interface protrusion is observed (see Fig. S8 in Supplementary Information), resulting in only a minor increase in channel cross-section and volume. The drag reduction performances that stem from the interface deflection alone are correspondingly low, amounting to only 1% for $Q=500\,\mu {{\rm{l}}}\,\min$-1, 1.6% and 2.7% for a flow rate of 750 and $1000\,\mu {{\rm{l}}}\,\min$-1, respectively. For the maximum flow rate, the channel enlargement leads to a change in pressure drop of 7.2 %.

In contrast, considering the meniscus interface as no-shear, it becomes evident that the pressure drop $\Delta {p}_{{{\rm{PSC}}}}^{{{\rm{sim}}}}$ over the 27 mm distance along the *z* − axis is significantly reduced, as illustrated in the last column of Table 2. The importance for the shear free characteristics of the interface is particularly evident at small meniscus protrusions. To illustrate, for $Q=500\,\mu {{\rm{l}}}\,\min$-1, the impact of the relatively modest volume increase is evidently outperformed by the slippery influence of the interface, namely by an additional 12%. For the maximum *Q*, the reduction in pressure drop due to the boundary condition is 18%, which attributes to 72% of the total drag reduction effect of 25%. We conclude that the drag reduction effect is predominantly influenced by the no-shear boundary condition at the meniscus interface.

## Discussion

A simple to fabricate partially substrateless microchannel (PSC) system was presented, realised as a large slit (width: 0.238 mm, length: 25 mm) integrated in one wall, to establish a stable air/liquid meniscus kept at atmospheric pressure during flow. The low Reynolds number (<10) for these flow conditions proves a steady and incompressible but non-uniform laminar flow. The non-uniformity arose from the varying radii of curvature along the flow direction owing to the pressure gradient driving the flow. Conventional pressure sensors were used to measure the pressure drop along the slit for a preset flow rate and thus to determine the overall drag reduction when compared with measurements of a reference microchannel (RC) with almost the same dimensions but with only no-slip solid walls. These flow experiments revealed a flow rate dependent maximum drag reduction of 25% while only 4% of the microchannel surface was altered.

During the flow experiments we observed, that the meniscus ruptured close to the beginning of the slit if the flow rate exceeded a critical value or what is equivalent if the local pressure exceeded the Laplace pressure drop across the meniscus interface. The maximum bounds of the local pressure were deduced from the geometry of the bulged meniscus and are in good agreement with experiments. However, the PSC exhibited self-healing properties. When the flow rate was aborted for some seconds, the meniscus recovered. After increasing *Q* again, the same drag reduction behaviour as before the rupture was observed. Additionally, we conducted flow experiments with a high flow rate for 24 h and observed no degradation of the drag reduction. These results also underline that the hydrophobic covalently bonded OTCS coating on the wall is stable.

Obviously, the two-point pressure sensor measurements are not adequate to study the interfacial dynamics in the slit area. Therefore, to investigate the mechanism of drag reduction of the PSC, we took advantage of the open access to the meniscus surface. Optical White Light Microscopy (WLI) was employed to image the complete topography of the meniscus. Profiles were extracted from these images. By fitting the data with a circular arc, it was possible to determine the radius of curvature and thus to evaluate the local static pressure distribution *p*_stat_(*z*) along the flow direction by applying the Young-Laplace equation. The static pressure values optically evaluated are in good agreement with the two local pressure sensor measurements. In fact, it was possible to detect smaller pressure values compared to the conventional pressure sensors. Limitations of this technique arise only if the meniscus curvature is to small in relation to the numerical aperture of the objective lens.

We like to emphasize that the optical measurement of the complete dependence *p*_stat_(*z*) gives the opportunity to identify the impact of the two main effects that influence drag reduction: the variation of the cross sectional area along *z* and the amount of the air/water surface with no-shear boundary condition. This was demonstrated performing hydrodynamic simulations with a commercial software package (COMSOL Multiphysics). Taking the curved topography from WLI measurements as geometrical input data for these simulations and assuming the meniscus surface as shear-free revealed that the main effect on drag reduction is due to the no-shear boundary condition while the enhancement of the cross sectional surface by meniscus bending is of minor importance.

## Methods

### Fabrication of top wall

The upper wall of the microchannel device has been constructed using a macroscopically structured silicon chip (width *w*_Si_ = 22.7 mm, length *l*_Si_ = 85.7 mm, thickness *t*_Si_ = 525 *μ*m) coated with a 1 μm thin silicon dioxide layer. For its processing rectangular areas of different sizes were opened in the silicon dioxide layer on both sides of the wafer with the openings arranged in superimposed configuration. Then silicon was dry etched from the backside using inductively coupled plasma reactive ion etching leaving a silicon membrane of 100 μm thickness. Subsequently, silicon was anisotropically dry etched from the front side, resulting in the formation of a slit with an open area of 25 mm in length and 0.238 mm in width. An additional isotropic dry etching process was employed to slightly underetch the silicon dioxide layer forming a SiO_2_ overhang structure of 3 μm width, serving to pin the meniscus. In the final step, the top wall was hydrophobised by immersing it in a 3 mM solution of OTCS (octadecyltrichlorosilane, Aldrich, purity  > 90%) in cyclohexane for 18 h (see details in Section S1 in Supplementary Information).

### Flow rate and pressure measurements

Flow experiments were carried out in a custom built microfluidic setup varying the flow rate with a flow rate contoller (BFS2, Elveflow) mounted close to the inlet port. The resulting pressure drop along the slit was measured with two pressure sensors (MPS0, Elveflow, accuracy ∓30 Pa) installed at *z* = − 1 mm and *z* = 26 mm (see Fig. 1). Using deionised water as the flow medium, we obtain a Reynolds number of up to 10, a capillary number of 10^−4^, and a Weber number of 10^−3^.

### White light interferometer

The surface topography of the meniscus in the slit area was imaged with a White Light Interferometer (NexView NX2, Zygo Ametek, spectral bandwidth: 350–750 nm, centre wavelength: 550 nm, *z*-accuracy approximately 1 nm) using a Mireau objective with ×10  magnification (NA = 0.30). One axis of the 2D scan area of the WLI was aligned in the flow direction. To record the meniscus over its full length, a stitching technique was used with an 80% overlap of the individual images, thereby minimising the travel distance between them to reduce the amount of vibration. The recorded data was then analysed using a custom written Python script to determine the radii of curvature along both the direction parallel and perpendicular to the flow direction.

### Numerical setup

To verify the experimental setup and subsequent measurement results, numerical calculations were performed using the commercial finite-element solver COMSOL Multiphysics®. We considered a laminar, incompressible and stationary duct flow, governed by the three-dimensional Navier-Stokes equations (see Section S9 in Supplementary Information).

## Supplementary information

Supplementary Information to Paper

## Data Availability

The data that support this study has been deposited in the repository accessible under 10.26204/data/8.
