# Supplementary material for: Partially substrateless microchannels for direct monitoring of interfacial dynamics in hydrophobic surfaces
Source: Commun Eng. 2025 Mar 13;4:46. doi: 10.1038/s44172-025-00386-6 (PMC11906875; doi:10.1038/s44172-025-00386-6)
Supplement: Supplementary file 1 — Supplementary Information to Paper [file 44172_2025_386_MOESM1_ESM.pdf]

Supplementary Information to Paper:

Partially Substrateless Microchannels for Direct Monitoring of Interfacial Dynamics in Hydrophobic Surfaces

Ellen Bold[a], Sebastian Zimmermann[b], Clarissa Schönecker[b], Egbert Oesterschulze[a],*

[a]*Rhineland-Palatinate Technical University (RPTU) Kaiserslautern, Department of Physics, Physics and Technology of Nanostructures, Erwin-Schrödinger Str. 46, Kaiserslautern, 67663, Rhineland-Palatinate, Germany*
[b]*Rhineland-Palatinate Technical University (RPTU) Kaiserslautern, Department of Mechanical Engineering, Microfluidics, Gottlieb-Daimler Str. 46, Kaiserslautern, 67663, Rhineland-Palatinate, Germany*

Content:

Section S1. Fabrication of samples
Section S2. Microchannel geometry and dimensions
Section S3. Validation and calibration of flow setup
Section S4. Meniscus profiles
Section S5. Local pinning angle
Section S6. Stability of meniscus
Section S7. Long-term stability of meniscus
Section S8. Limits for the detection of the meniscus with the WLI
Section S9. Numerical setup for flow simulations

S1. Fabrication of samples

The key component of our microchannel is a macroscopically structured silicon chip (width $w_{\mathrm{Si}} = 22.7\,\mathrm{mm}$, length $l_{\mathrm{Si}} = 85.7\,\mathrm{mm}$, thickness $t_{\mathrm{Si}} = 525\,\mu\mathrm{m}$) used as top channel plate. They are made from silicon wafers

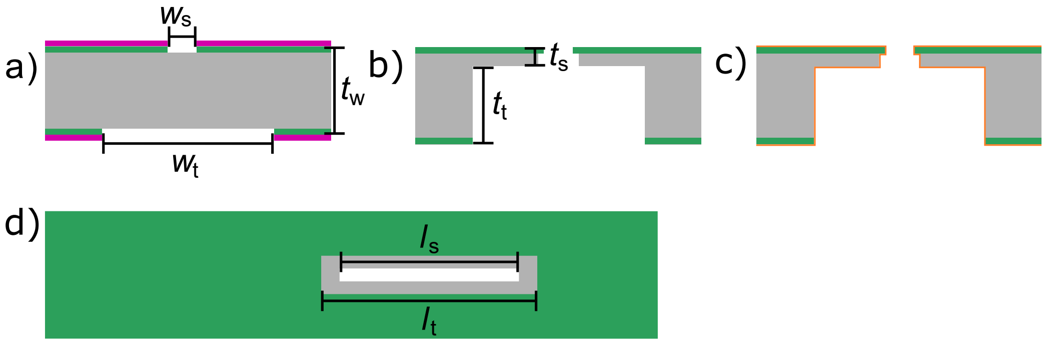

Fig. S1: Scheme showing profiles and top view of the silicon chip: a) silicon chip (grey, thickness t_{Si} = 525 μm) was thermally oxidized to receive a 1.0 μm thin layer of SiO_2 (green) and subsequently coated with a photoresist layer (purple, thickness 1.4 μm). Both layers were structured to form a slit (width $w_s = 0.238$ mm) on top and a trench (width $w_t = 4.5$mm) on bottom. b) Chip after ICP RIE etching of silicon from both sides of the wafer forming the slit in the silicon membrane ($t_s = 100\,\mu$m, $t_t = 425\,\mu$m). c) The slightly underetched silicon dioxide layer is finally coated with a monolayer of OCTS (yellow). d) Top view of the chip from the back side showing the open slit (length $l_s = 25$ mm) centred on the silicon membrane (length $l_t = 27$ mm) without OTCS layer. Dimensions are not to scale.

(4 inch diameter, (100) crystal orientation, double-sided polished, thickness $t_{Si} = 525\,\mu$m). Scheme of the fabrication process is shown in Fig. S1. The wafers were first oxidised to obtain a 1 μm thin silicon dioxide layer. Both the bottom and top surface were then spin coated with a 1.4 μm thin hydrophobic UV photo resist layer (AZ1512 HS) using a thin layer of hexamethyldisilizane as an adhesion promoter. UV laser lithography (Microtech LW405D) was applied to expose rectangular areas of different sizes on both sides adjusting them on top of each other. After removal of the exposed photoresist, the silicon dioxide layer in these areas was etched off with an aqueous solution of buffered HF (Sigma-Aldrich BOE 6:1). Subsequently, inductively coupled plasma reactive ion etching (ICP-RIE) of silicon was performed on the backside using the photoresist coated silicon dioxide layer as hard mask layer until a depth of approximately $t_t = 425\,\mu$m was reached forming a $l_t = 27$ mm long and $w_t = 4.5$ mm wide trench. The same process was applied to the top of the wafer until the silicon in the slit area was completely removed. Then an isotropic dry etching process was applied for only 20 s to slightly underetch the SiO_2 layer. Thus after removal of the photoresist layer an approximately 3 μm wide released layer of silicon dioxide was obtained, forming the rim

area of the 25 mm long and 0.23 mm wide slit. During channel flow, this thin layer constitutes an overhang structure that is advantageous for the stability of the meniscus.

The stability of the meniscus has been improved by depositing a hydrophobic film on the silicon dioxide layer as well as on all silicon surfaces that have a thin native silicon dioxide layer that forms during exposure in air. Prior to the surface coating process, the wafer was thoroughly cleaned by sonication in acetone, isopropanol, and water to remove photoresist residues. The surface was then activated by VUV light exposure (ExciJet172 55-130, wavelength 17.6 nm) for several minutes. Finally, the chip was immersed in a 3 mM solution of OTCS (octadecyltrichlorosilane, Aldrich, purity $> 90\,\%$) for 18 h forming a self-assembled hydrophobic OCTS layer. The wafers were rinsed with toluene and deionised water and dried under a stream of dry nitrogen (Fig. S1 d)).

Additionally, another silicon chip was fabricated leaving the flat silicon surface unstructured. Installed in the microchannel stack, a conventional microchannel with a rectangular cross-section is formed, with the walls fulfilling the no-slip boundary condition. It is used as reference channel (RC) when performing flow experiments with the PSC.

S2. Microchannel geometry and dimensions

The channel height h_{c} has a large influence on the correct estimation of λ_{eff} and the correct geometry for the simulation. Therefore, before and after each measurement, the area between the pressure sensor connectors, which can be seen as black holes (Fig. S2), was imaged with the 50$\times$ objective of the WLI. In addition, the step height between the lower side of the SiO_2 overhang layer and the PMMA top surface was measured prior to the start of the flow experiment to ensure that the film was not deformed during the assembly of the microchannel. A consistent height of 176.9 μm was obtained with a maximum standard deviation of 1.1 μm along the 27 mm long microchannel. The width $w_{\mathrm{c}} = 238 \pm 1\,\mu$m and length $l_{\mathrm{c}} = 25,000 \pm 10\,\mu$m of the slit were accordingly measured.

S3. Validation and calibration of flow setup

The reference sample was used to validate the measurement setup assuming no-slip boundary condition at the walls of the microchannel. To consider

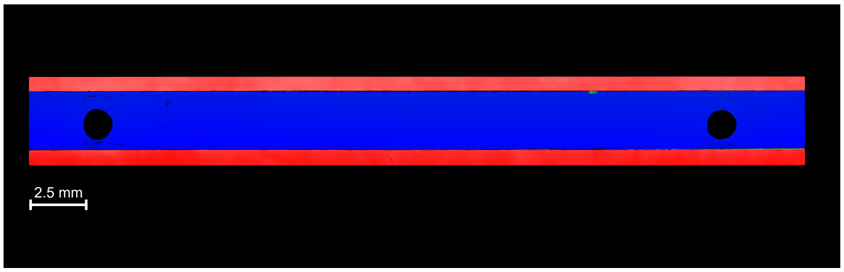

Fig. S2: Stitched WLI depth image (1,800 × 16,000 pixel) of the area close to the pressure sensor connectors (shown by the two black holes). The height h_c between the sealing sheet (red) and the PMMA top surface (blue) is $176.9 \pm 1.1\,\mu$m.

the influence of temperature fluctuations in the laboratory, measurements were performed before and after each flow experiment showing a deviation of maximum 2 Pa. This is well below the specific resolution of ±30 Pa of the pressure sensors (Microfluidic Pressure Sensor MPS0, Elveflow).

The pressure sensor measurements for varying flow rate Q were then compared with the theoretically calculated pressure drop $\Delta p^{\mathrm{theo}}_{\mathrm{no-slip}}$, as outlined in Sec. 2.2 of the paper. The experimental and theoretical results are consistent with each other, indicating a proper working experimental setup.

S4. Meniscus profiles

Meniscus profiles (50 of approx. 28,600 recorded profiles) with a distance of $\Delta z = 0.5$ mm to each other are shown in Fig. S3 for each flow rate Q investigated. With increasing Q and thus increasing local pressure $p_{\mathrm{stat}}(z)$, the meniscus curvature at a given location z is increasing. This is consistent with the fact that the static pressure is given by the Laplace pressure which scales with $1/R_{\perp}(z)$ (see Sec. 2.3 in the paper).

For Q lower than 1,250 μl min-1 the linear decrease of the height profiles (blue crosses) is evident. Only for $Q = 1,500\,\mu$l min-1 fluctuations are observed, which are insignificant when the height variations of maximum 10 μm are related to the length scale of 5 mm. They are in particular observed, when the flow rate is just below the critical value Q_{crit} where rupture of the meniscus may occur.

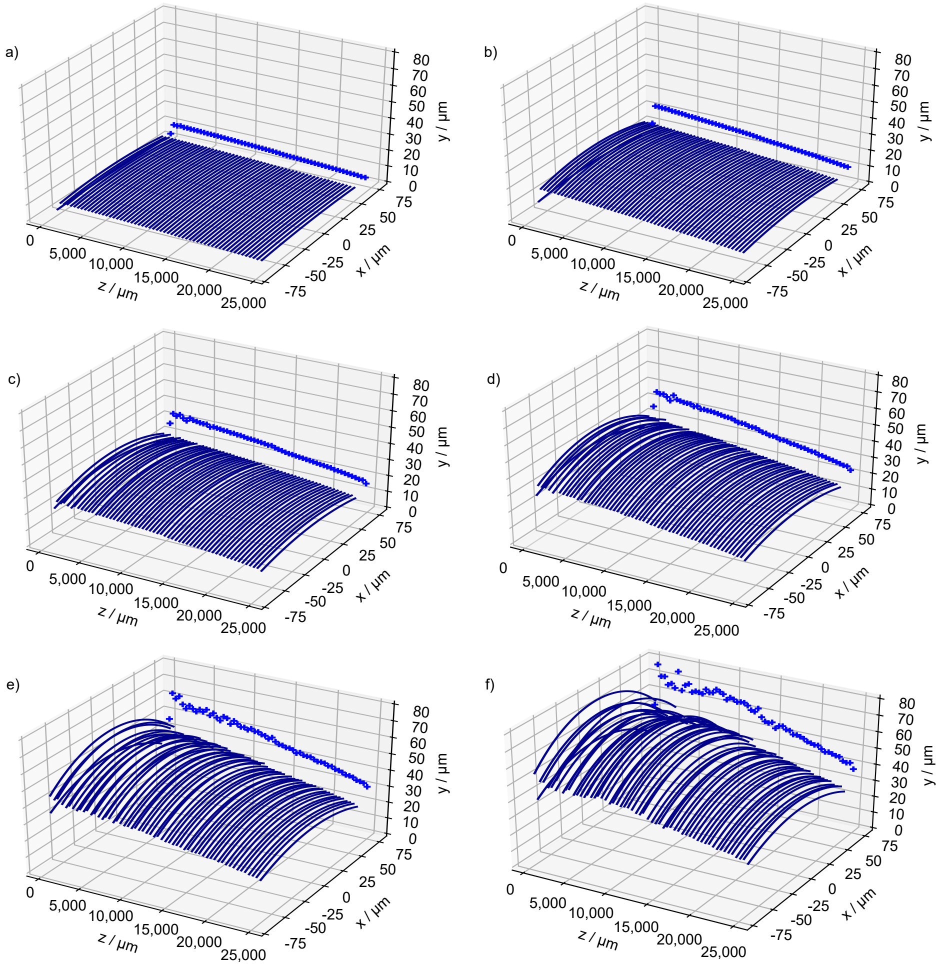

Fig. S3: Meniscus profiles perpendicular to the flow direction were extracted from WLI measurements of its surface within the imaging constraints. Adjacent profiles have a separation distance of $\Delta z = 0.5\,\text{mm}$. The maximum profile height $h(z)$ was additionally projected onto the $y - z$ plane (blue crosses). Profiles are shown for flow rates Q: a) $250\,\mu\text{l min-1}$, b) $500\,\mu\text{l min-1}$, c) $750\,\mu\text{l min-1}$, d) $1{,}000\,\mu\text{l min-1}$, e) $1{,}250\,\mu\text{l min-1}$, and f) $1{,}500\,\mu\text{l min-1}$.

S5. Local pinning angle

The local angle $\theta(z)$, at which the meniscus pins at the edges of the overhang silicon dioxide layer, can be obtained extrapolating the local WLI profiles to the edge of the overhang structure. We observe that $\theta(z)$ increases almost linearly along the z-axis for a given value of Q (Fig. S4). Increasing

the inlet pressure leads to an increase of both Q and the local pressure $p_{\mathrm{stat}}(z)$, which is given by the Laplace pressure. Therefore, we expect the local radius of curvature and thus the local pinning angle $\theta(z)$ also to increase with increasing Q which is consistent with the experimental data for three different Q values shown in Fig. S4.

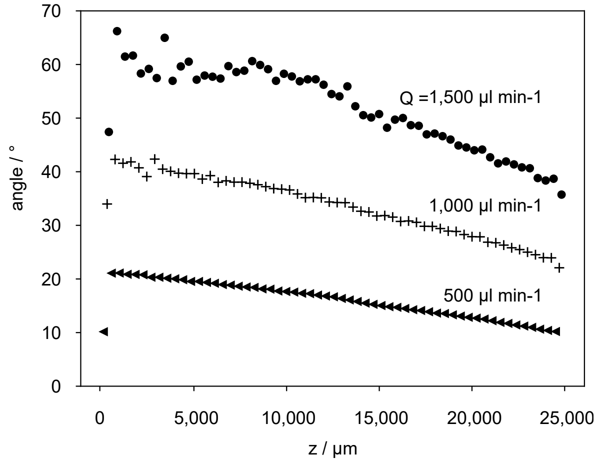

Fig. S4: The spatially resolved pinning angle $\theta(z)$ obtained at the edge of the overhang structure by extrapolation of evaluated profiles for three different flow rates.

S6. Stability of meniscus

Figure S5 shows the cross section of the meniscus pinned at the upper edges of the overhang structure on both sides of the slit of the width w_{c}. If the pinning angle $\theta(z)$ exceeds the critical angle θ_{crit}, the meniscus ruptures aborting water transport through the microchannel. Although an advancing angle of a water droplet on a flat OTCS layer of 114° was measured, we observe in the experiment, that the meniscus burst already for a pinning angle close to 70°.

To understand this discrepancy, we evaluate the scheme in Fig. S5. From the triangle ΔABM we get:

$$\cos(\theta_{\mathrm{crit}} - 90°) = \sin(\theta_{\mathrm{crit}}) = \frac{w_{\mathrm{c}}}{2R_{\mathrm{min}}}, \tag{1}$$

where R_{min} is the minimum radius received for a stable meniscus at the critical angle θ_{crit}. Solving the last equation for R_{min} and entering it into

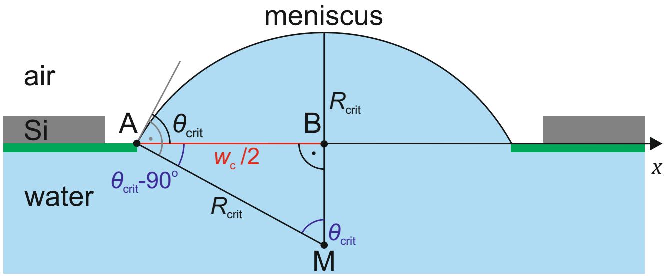

Fig. S5: Schematic cross section through the upper wall of the microchannel. The meniscus pins at the upper edges of the OTCS coated SiO_2 overhang layer on each side of the slit with width w_c. The meniscus profile forms a circular arc of minimum radius R_{min}. If $\theta(z)$ approaches θ_{crit}, the meniscus becomes unstable and ruptures.

Laplace's equation gives an expression for the maximum possible local pressure the meniscus can withstand:

$$\Delta p_{\text{Laplace}}^{\text{max}} = \sigma / R_{\text{min}} = \frac{2\sigma \sin(\theta)}{w_{\text{c}}}. \qquad (2)$$

With $w_c = 238\,\mu m$, $\sigma = 0.072\,\text{N m-1}$ for DI water (MilliQ, resistivity: 18.2 MΩcm), and $\theta_{adv} = 114°$ for DI water on the flat OTSC layer, the maximum calculated pressure drop across the meniscus is about 573 Pa. This calculated value is in good agreement with the measured pressure value of sensor PS1 of approx. 600 Pa for the maximum flow rate of $Q = 1,500\,\mu\text{l min-1}$ when this pressure value is corrected for the 1 mm distance between the location of the pressure sensor and the location where the first WLI profile is evaluated.

However, in the experiments we observe a critical angle of only $\theta_{crit} \approx 70°$ for the above evaluated pressure. This discrepancy of 44° for the critical angle stems from the fact that the last equation has two solutions for the critical angle θ_{crit} for a given critical pressure value. They are located symmetrically with respect to the first maximum of the sine function at an angle of 90°. This means if the pinning angle of the bulging meniscus reaches the first solution at 70° the meniscus already bursts exactly at the pressure calculated above. As $\sin(70°) \approx \sin(114°)$, it is impossible to observe the larger angle of 114° in the experiment.

S7. Long-term stability of meniscus

The meniscus microchannel device was operated for 24 h presetting the flow rate to 1,000 μl min-1 to investigate in particular the stability of the

meniscus and the resultant flow properties. During this period, the pressure drop measured continuously with the pressure sensors was stable and small fluctuations due to temperature variations lead to a standard deviation of 3.5% (Fig. S6). These results underline that the meniscus is stable under the imposed flow rate and does not lose its stability over time. This also indicates that the OTCS layer does not degrade on this time scale.

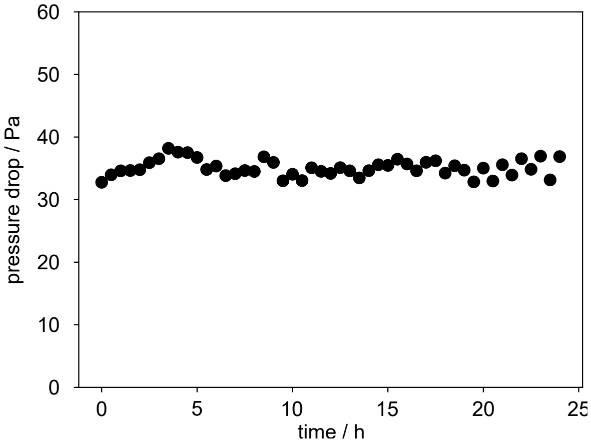

Fig. S6: Pressure drop measurements over 24 h of the meniscus sample demonstrating its long term stability.

S8. Limits for the detection of the meniscus with the WLI

A light cone of opening angle 2ϑ emerges from the objective lens of the WLI and illuminates the meniscus surface where ϑ is defined by the numerical aperture (NA) of the objective lens with NA$=$ n $\sin(\vartheta)$ (see Fig. S7). Here, the refractive index of the immersing medium (air) is assumed to be 1.0. When this light cone is reflected by positioning the objective lens at the maximum height of the droplet (position 1 in Fig. S7), almost all the rays of the cone of light are reflected back into the entrance pupil of the objective lens. However, if the objective lens is scanned to position 2 to the left (or right) by Δx, the amount of light passing through the entrance pupil after reflection at the meniscus surface is continuously reduced for increasing Δx. Finally, for scan lengths greater than:

$$\Delta x_{\max} = R \cdot \sin(2\vartheta)$$

no more light enters the entrance pupil (see Fig. S7). Therefore, we only get information about the meniscus topography in the scan range from $-\Delta x$ to $+\Delta x$. If Δx_{max} exceeds $w_c/2$, the WLI is able to image the entire meniscus profile. Otherwise only the central part of the profile is imaged.

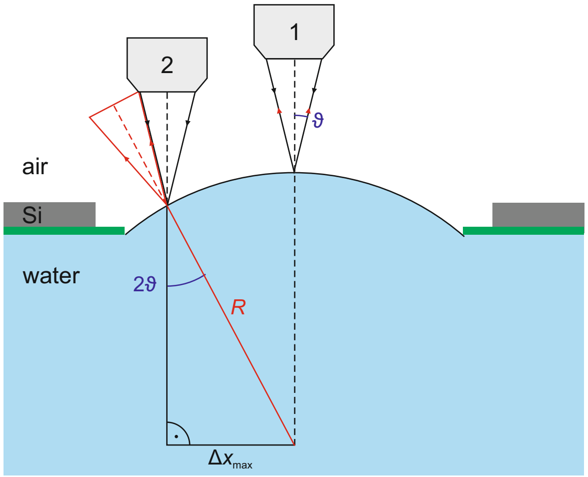

Fig. S7: Geometry for rays emerging from the WLI objective lens that are reflected back from the meniscus surface for the objective lens located at position 1 at the centre of the slit corresponding to the position of the maximum height of the meniscus and 2 a position that is translated by Δx with respect to position 1 along x.

When we consider to resolve the complete meniscus profile with $\Delta x = w_c/2$, then the minimum radius R_{min} that can still be evaluated is given by:

$$R_{\text{min}} = \frac{w_c}{2 \cdot \sin(2\vartheta)}.$$

In Tab. S1 the minimal detectable radius R_{min} was collected for the objective lenses delivered with the WLI from Zygo.

To resolve the full meniscus profiles independent of the flow rate Q, it is crucial to use the 50× objective as can be deduced from the minimum radius of 121 μm occurring in the experiments (see Fig. 4 d) in the paper). In the experiment, however, the stitching of the 25 mm long meniscus topography required the acquisition of up to 100 WLI images. Therefore, to keep the measurement time acceptable, we have used the 10× objective lens (one measurement takes 20 min). It allows to resolve the central part of each profile. This is sufficient because these profiles resemble circular arcs that

Tab. S1: Collected data of the minimal detectable radius of curvature R_{min} for our PSC with the available objectives lenses of the Zygo WLI, when the full meniscus profile must be resolved.

Magnification	NA	R_{min} in μm
10×	0.30	208
20×	0.40	162
50×	0.55	129

can be easily evaluated to extract the local radius $R_{\perp}(z)$ (see Sec. 2.3 in the paper) and thus the local pressure $p_{\mathrm{stat}}(z)$ that we are interested in to evaluate the local flow conditions in the microchannel.

S9. Numerical setup for flow simulations

To verify the experimental setup and subsequent measurement results, numerical calculations were performed using the commercial finite-element solver COMSOL Multiphysics®. We consider a laminar, incompressible and stationary duct flow, governed by the three-dimensional Navier-Stokes equations

$$\rho\left(\boldsymbol{u}\cdot\nabla\right)\boldsymbol{u} = -\nabla p + \mu\nabla^2\boldsymbol{u}, \tag{3}$$

with the velocity vector $\boldsymbol{u} = (u, v, w)$, the pressure-drop Δp, the Nabla operator $\nabla = \left(\frac{\partial}{\partial x}, \frac{\partial}{\partial y}, \frac{\partial}{\partial z}\right)$ in Cartesian coordinates, and the dynamic viscosity μ and density ρ of deionised water, respectively.

Figure 6 a) in the paper shows an example half-width duct layout, assuming an overall channel width of $w_{\mathrm{c}} = 2.5\,\mathrm{mm}$, a channel height of $h_{\mathrm{c}} = 0.176\,\mathrm{mm}$, and a channel length of 27 mm.

A fully developed velocity field is implemented as inlet boundary condition located 2 mm in front of the slit (corresponding to $z = -2\,\mathrm{mm}$), calculated on base of the experimentally determined volume flux of 500 to 1,500 μl min-1 in steps of 250 μl min-1 for each study. Furthermore, we choose a pressure-outlet $p = 0\,\mathrm{Pa}$ located 2 mm after the slit and implement a symmetry condition along the right-hand-side boundary. No potential mobility reduction of the interface due to deposited surfactants or impurities were considered.

To model the shape of the meniscus for each case considered, the radius of curvature at $z = 961\,\mu\mathrm{m}$ measured by the WLI was first initialised as a

circular arc (x, y)-cross-section of channel enlargement. The progression of interface protrusion was then assumed to linearly decrease along the z-axis. However, to account for the fact that the interface pins at the edge of the silicon dioxide overhang structure, the meniscus protrusion was interpolated quadratically down to zero at $z = 0\,\mathrm{mm}$ and $z = 25\,\mathrm{mm}$, or in other words, at the start and end of the slit. Crucially, we do not explicitly considered the air-phase in our numerical setup. Instead, the water-air interaction along the meniscus was modelled as a shear-free boundary, thereby enabling the bulk flow to slip along these regions without experiencing any resistance (a (x, y)-cross-section of a meniscus is illustrated as a dashed line in Fig. S8). Although the air is modelled to be non-viscous, our approach has been proven by numerous studies to be a quite accurate approximation to model air-water interface interaction [24]. The triangular mesh has been highly refined along the interface to adequately resolve these connection conditions. All remaining surfaces are defined as no-slip solid walls, meaning that both the tangential and normal velocity components disappear. A mesh study was conducted to ensure that the results were not affected by the mesh-size.

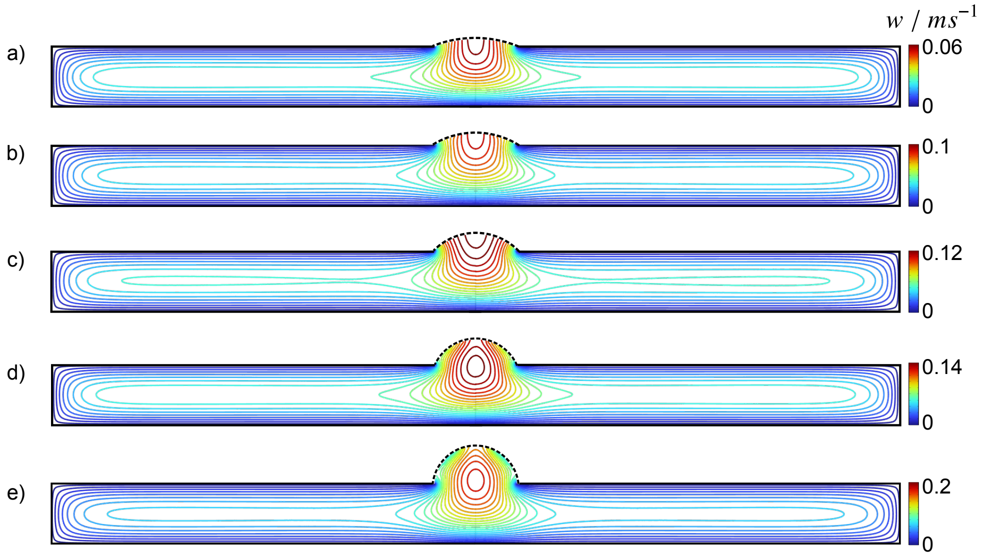

Fig. S8: Velocity field contour-line plots in the (x, y)-cross-section at $z = 1\,\mathrm{mm}$ for different flow rates Q: a) $500\,\mu\mathrm{l\,min}$-1, b) $750\,\mu\mathrm{l\,min}$-1, c) $1,000\,\mu\mathrm{l\,min}$-1, d) $1,250\,\mu\mathrm{l\,min}$-1, and e) $1,500\,\mu\mathrm{l\,min}$-1. Each plot has its individual colour scale as shown by the colour bar. Black dashed lines show the protruded menisci in each case.
